# Feasibility of Social Media–Based Recruitment and Perceived Acceptability of Digital Health Interventions for Caregivers of Justice-Involved Youth: Mixed Methods Study

**DOI:** 10.2196/16370

**Published:** 2020-04-30

**Authors:** Johanna Bailey Folk, Anna Harrison, Christopher Rodriguez, Amanda Wallace, Marina Tolou-Shams

**Affiliations:** 1 Department of Psychiatry University of California San Francisco, CA United States; 2 San Francisco Veterans Affairs Medical Center San Francisco, CA United States; 3 New York-Presbyterian/Columbia University Irving Medical Center New York, NY United States

**Keywords:** caregivers, telemedicine, mobile health, juvenile delinquency, social media

## Abstract

**Background:**

Caregiver involvement is critical for supporting positive behavioral health and legal outcomes for justice-involved youth; however, recruiting this population into clinical research studies and engaging them in treatment remain challenging. Technology-based approaches are a promising, yet understudied avenue for recruiting and intervening with caregivers of justice-involved youth.

**Objective:**

This mixed methods study aimed to assess the feasibility of recruiting caregivers of justice-involved youth using social media into clinical research and to understand caregivers’ perceptions of the acceptability of digital health interventions.

**Methods:**

Caregivers of justice-involved youth were recruited through paid Facebook advertisements to participate in a Web-based survey. Advertisement design was determined using Facebook A/B split testing, and the advertisement with the lowest cost per link click was used for the primary advertisement campaign. Survey participants were offered the option to participate in a follow-up qualitative phone interview focused on the perceived feasibility and acceptability of digital health interventions.

**Results:**

Facebook advertisements were successful in quickly recruiting a diverse set of caregivers (80/153, 52.3% female; mean age 43 years, SD 7; 76/168, 45.2% black, 34/168, 20.2% white, and 28/168, 16.7% Latinx; and 97/156, 62.2% biological parents); cost per click was US $0.53, and conversion rate was 11.5%. Survey participants used multiple social media platforms; 60.1% (101/168) of the participants indicated they would participate in a digital health intervention for caregivers of justice-involved youth. Survey respondents’ most preferred intervention was supportive and motivational parenting messages via SMS text message. Of the survey respondents, 18 completed a phone interview (12/18, 67% female; mean age 45 years, SD 10; 10/18, 56% black, 7/18, 39% white, and 1/18, 6% Latinx; and 16/18, 89% biological parents). Interview participant responses suggested digital health interventions are acceptable, but they expressed both likes (eg, alleviates barriers to treatment access) and concerns (eg, privacy); their most preferred intervention was video-based family therapy.

**Conclusions:**

Recruiting and intervening with caregivers of justice-involved youth through social media and other digital health approaches may be a feasible and acceptable approach to overcoming barriers to accessing traditional in-person behavioral health care.

## Introduction

### Background

Each year in the United States, more than 920,000 youth under the age of 18 years are arrested [1], and juvenile courts process nearly 1 million delinquency cases [2]. Involvement in the juvenile justice system is associated with various adverse outcomes, including substance use [3], psychiatric disorders [4], and sexual risk behavior [5,6]. Family factors (eg, parent-child conflict and coercive parenting) are consistently associated with youth delinquency [7-10]. As family-based interventions effectively improve outcomes among justice-involved adolescents [11-13], involving family members, particularly primary caregivers, in therapeutic interventions may be an effective way to support positive youth outcomes [14].

Despite the importance of caregiver involvement, recruiting broad samples of caregivers into clinical research remains challenging. Caregivers are typically *not* court ordered to treatment along with their youth. Resources required to attend treatment (eg, time and reliable transportation) may be scarce. Families often feel overwhelmed by the number of required services related to their youth’s court involvement (eg, court appointments, drug screens, and mandated treatment) [15]. Therefore, caregiver treatment attendance is often poor, and study attrition for clinical trials of family-based interventions is high [16]. The most effective behavioral health treatments include caregivers [17,18], but engaging face-to-face with researchers and clinicians may not be feasible.

New strategies are needed to engage caregivers of justice-involved youth into clinical research and treatment. Technology offers multiple promising avenues. In particular, digital mobile health (mHealth) technology is an efficacious, low-cost way of reaching underserved, vulnerable populations to engage them into and/or deliver quality care [19]. mHealth circumvents many barriers to treatment participation reported by caregivers of justice-involved youth [20], allowing for instantaneous, portable access and direct communication with providers [21]. mHealth effectively increases patient communication, monitoring, and education to reduce the burden of diseases associated with poverty and improves access to health services, clinical diagnosis, and treatment adherence [22]. Social media, specifically Facebook, is one mHealth approach that has been successful in recruiting and intervening with hard-to-reach populations [23-25], including sexual minorities [26] and young adult veterans [27].

Caregivers of justice-involved youth are interested in mHealth treatment [28,29], although little is known about what specific approaches would be most acceptable and beneficial for them. One pilot study with 5 caregivers of justice-involved youth found preliminary evidence for the feasibility and acceptability of a text messaging intervention that sent appointment reminders and motivational messages [29]. To our knowledge, no prior study has used social media to engage or intervene with caregivers of justice-involved youth. Thus, little is known about (1) the feasibility of engaging caregivers of justice-involved youth in clinical research using social media and (2) whether caregivers would consider participating in a range of digital health interventions. Engaging caregivers in clinical research and treatment through mHealth technology may expand access to family-based treatments and thereby promote health equity.

### This Study

This mixed methods study aimed to assess the feasibility of using social media to recruit caregivers of justice-involved youth into research studies and to understand caregivers’ perceptions of the acceptability of digital health interventions. Caregivers were recruited through Facebook to complete a Web-based survey and an optional follow-up phone interview. We hypothesized Facebook would be a feasible recruitment tool, and caregivers would be open to participating in a range of digital health interventions. The overall goal was to lay the groundwork for future studies on delivering caregiver-focused and family-focused interventions through digital health platforms such as social media.

## Methods

### Participants and Procedure

#### Advertising Strategy

Facebook advertisements were optimized for link clicks and presented to adults (1) ages 28 years and older, (2) using Facebook in English, and (3) residing in 10 US metropolitan areas. The maximum daily budget was US $80 per day. See Multimedia Appendix 1 for additional details regarding pilot testing of advertisements and advertising strategy.

#### Quantitative Survey

Participants were a convenience sample of 168 caregivers of justice-involved youth. Eligible participants were (1) the caregiver of a youth (ages 10–17 years) who had been arrested, detained, or court involved during the past 12 months; (2) had access to a computer or mobile device with the internet; and (3) proficient in English.

Participants were recruited through Facebook Ad Manager. Advertisements were direct promotions for the survey website; individuals who clicked the advertisement were directed to an external website (Research Electronic Data Capture) containing a Web-based screening questionnaire. Eligible participants were directed to a study information page containing the consent form. A Facebook page where participants could review this material was maintained and reachable from the advertisements. Those who provided informed consent were directed to the Web-based survey. The open survey was presented across 10 screens with nonrandomized items and a varying number of items per page (influenced by skip logic); participants were free to skip any items and could not review or change answers from prior pages. To receive the US $15 electronic Amazon gift card incentive, participants entered an email address which, after checking to prevent multiple entries from the same individual, was disconnected from survey responses. We set a predetermined sample size goal of 150 participants based on available resources. All study procedures were approved by the University of California, San Francisco, Institutional Review Board (#18-25987).

#### Qualitative Interview

At the end of the survey, participants were asked if they would be willing to participate in a follow-up phone interview. Those who agreed (54/168, 32.1%) provided contact information, which was immediately disconnected from survey responses; 18 qualitative interviews were completed, at which point saturation (ie, when no new themes arose) had been reached [30]. Most participants expressing interest in the phone interview identified as female, so quota sampling (ie, aims to create a sample that represents certain characteristics of a population [31]) was used to ensure adequate male representation. Interviews were approximately 45-min long and participants received a US $50 electronic gift card.

### Measures

#### Quantitative Survey

##### Sample Characteristics

Caregivers identified their own and their justice-involved youth’s gender, race, ethnicity, and marital status.

### Youth Justice Involvement, Behavioral Health Needs, and Treatment

Caregivers reported their youth’s past year justice involvement (eg, probation and detention), behavioral health needs (eg, mental health diagnosis), and participation in behavioral health treatment.

### Technology and Social Media Usage

Caregivers reported their access to technology (eg, smartphone and computer) and social media (eg, Facebook and Twitter) use.

### Acceptability of Digital Health Interventions

Caregivers reported their willingness to participate in digital health interventions for caregivers of justice-involved youth (1=*I would definitely not participate* to 5=*I would definitely participate*). Participants were asked whether they would participate in any of the following interventions and to rate which three were most interesting: (1) receiving supportive/motivational parenting messages via SMS text message; (2) supportive/motivational parenting posts on social media platforms (eg, Instagram and Snapchat); (3) online support community; (4) private groups on Facebook connecting caregivers with one another; (5) private groups on Facebook connecting caregivers and where they can chat with/contact a mental health professional; (6) individual therapy sessions with a therapist through video chat (eg, FaceTime and Skype); (7) family therapy sessions, including the caregiver, their child, and other family members, through video chat; (8) support group meetings with other caregivers through video chat; and (9) other (please specify).

#### Qualitative Interview

In-depth semistructured phone interviews were conducted in a private research laboratory by the first (JF) and third (CR) authors, who were trained and experienced in qualitative interviewing. Descriptive phenomenological interviews [32] asked participants about the (1) impact of their youth’s justice involvement on different aspects of their lives (eg, work and relationships), (2) family engagement in behavioral health services as recommended or mandated by the court, (3) technology and social media use, and (4) opinions of three hypothetical digital health interventions (primary outcome). The semistructured interview format was selected to allow respondents to express complex thoughts without restriction and to allow interviewers to probe when clarification and/or depth was necessary [33]. All interviews were audio recorded using a digital tape recorder and transcribed nonverbatim, excluding nonverbal cues for a more comprehensible transcript. To ensure the accuracy of the transcript, one researcher (CR) reviewed each transcript while listening to the audio recording.

### Sample Characteristics

Caregivers reported their gender, age, race, ethnicity, marital status, and relationship to the justice-involved youth. They also reported their youth’s age and current justice involvement.

### Technology and Social Media Usage

Caregivers reported their access to technology, most frequently used social media platform, reasons for using social media (eg, connect with family), and online support group participation.

### Digital Health Intervention Vignettes

Participants were presented with three vignettes of hypothetical digital health interventions during the qualitative interview. They included (1) private groups on Facebook connecting caregivers of youth involved in the justice system with one another with the option to chat with/contact a mental health professional; (2) family therapy sessions, including the caregiver, their child, and other family members, through video chat; and (3) receiving appointment reminders and supportive/motivational parenting messages via SMS text message. Participants were asked to share their likes and dislikes about each intervention and any potential barriers to or benefits from participating.

### Coding and Analysis

Inductive Thematic Analysis was selected as the analytic approach to allow themes to emerge from raw data and diminish researcher bias [34]. The initial coding scheme was informed by the semistructured interview guide and interview transcripts, ensuring the authenticity of the participant’s perspective. Before data analysis, three researchers (JF, CR, and AW) employed a co-coding pilot with three randomly selected interview transcripts. Codes from the initial coding scheme were independently applied to interview transcripts and then compared to address inconsistencies (eg, different codes applied to the same section and interpreting the same code differently) and track similarities. The coding scheme was finalized after four iterations of the co-coding pilot. Revising ambiguous codes to improve intercoder reliability [35,36] preserved the integrity of the data and enhanced the rigor of the study.

On completion of the co-coding pilot, the three coders independently coded the remaining 15 interview transcripts; each transcript was coded by two of the researchers, and any disagreements were discussed to achieve a final agreement code. All interview transcripts were coded using Atlas.ti 8.0 (ATLAS.ti Scientific Software Development GmbH, Germany) [37]. Memos were written after various coding iterations to continuously develop assertions (ie, claims about the data supported by direct evidence) and propositions (ie, inferences that provide potential relationships or possible explanations of the explored theory) and again after all interviews were coded to organize common themes among and relationships between data [38]. Memoing further enhanced rigor by allowing researchers to reflect on biases that could influence the participant’s perspective and make adjustments accordingly.

## Results

### Facebook Advertisements

Pilot testing ran from November 4, 2018, to November 8, 2018 (4 days). Advertisements were shown to 37,630 Facebook users, resulting in 461 unique link clicks. A total of US $318.08 was spent on advertisement pilot testing. Cost per click varied by advertisement (see Multimedia Appendix 1), with the most successful advertisement (woman comforting a teenager with his head down) costing US $0.47 per click and the least successful (juvenile detention center) costing US $0.91 per click.

The single most successful advertisement was used for the primary campaign. The campaign was funded from November 10, 2018, to December 3, 2018 (23 days), at which point we surpassed our target sample of 150 caregivers. Advertisements were presented to 500,208 Facebook users, and of those, 3394 clicked the advertisement link. The cost per click was US $0.53. Of the users who clicked the advertisement, 389 completed the Web-based screener (11.5% conversion rate), 235 were eligible, 185 consented to participate, and 168 completed the survey. The total primary campaign advertising cost was US $1802.72, which translated to an advertising cost of US $10.73 per participant.

### Quantitative Survey

#### Sample Characteristics

Caregivers were 52.3% (80/153) female, on average 43 years old (SD 7), and racially/ethnically diverse (76/168, 45.2% black; 34/168, 20.2% white; 28/168, 16.7% Latinx; and 26/168, 15.5% other). Caregivers were biological parents (97/156, 62.2%), step-parents (32/156, 20.5%), or nonfamilial foster (2/156, 1.3%) parents to predominantly male (109/159, 68.6%) justice-involved youth who were on average, 15 (SD 1) years old. (*N*s differ throughout results based on missing data.)

#### Youth Justice Involvement, Behavioral Health Needs, and Treatment

During the past year, youth were arrested twice on average (SD 2, range 1-10); 79.2% (122/154) youths appeared in court related to their arrest, and 67.1% (104/155) of youth were found delinquent by the court. During the past year, 48.1% (74/154) of youth spent time in a juvenile detention center or court-ordered residential placement, 45.1% (69/153) of youth were on probation, 23.2% (35/151) of youth were on electronic monitoring, and 16.4% (24/146) of youth had their case transferred from juvenile to adult court.

Of 151 caregivers, 54 (35.8%) reported they had ever been told their justice-involved youth had a mental health diagnosis. The most commonly reported diagnoses were attention deficit hyperactivity (23/168, 13.7%), depressive (19/168, 11.3%), anxiety (17/168, 10.1%), and bipolar (13/168; 7.7%) disorders. Posttraumatic stress (9/168, 5.4%) and substance use (4/168, 2.4%) disorders were reported at lower rates. Behavioral health treatment utilization was high, with 67.5% (104/154) of caregivers reporting their youth had ever participated in any type of behavioral health treatment (eg, psychiatric medication, residential treatment, and crisis center). More than one-third (50/133, 37.6%) of caregivers reported their youth had ever received private professional help from a psychiatrist, psychologist, social worker, or psychiatric nurse; of the 54 caregivers who had ever been told their youth had a mental health diagnosis, 27 (50%) of these youth had ever received private professional help from a psychiatrist, psychologist, social worker, or psychiatric nurse. School-based counseling was the most commonly used service (53/127, 41.7%), followed by psychiatric medication (37/146, 25.3%), community mental health centers (25/134, 18.7%), in-home counseling (25/135, 18.5%), therapeutic foster care (23/140, 16.4%), and residential treatment centers (21/140, 15.0%). Outpatient drug or alcohol clinic services were sought by 10.9% (15/138) of youth, and 14.0% (20/143) of youth had a history of inpatient alcohol/drug treatment or detoxification unit.

#### Technology and Social Media Usage

Most caregivers owned a smartphone/tablet (160/167, 95.8%) and had regular computer access (145/167, 86.8%). Caregivers endorsed using multiple social media platforms, most commonly Facebook (131/168, 78.0%), Instagram (107/168, 63.7%), YouTube (104/168, 61.9%), and Twitter (83/168, 49.4%). Snapchat, Pinterest, LinkedIn, and WhatsApp were each used by less than 35%; 2.4% (4/168) of caregivers reported they did not currently use social media. Of 156 caregivers, 44 (28.2%) were participating in an online support group and 58% (22/38) of those reported the group was specifically for caregivers of justice-involved youth.

#### Acceptability of Digital Health Interventions

Most caregivers were open to participating in digital health interventions, with 60.5% (101/167) caregivers indicating they would *probably* or *definitely* participate in an online intervention specifically. When asked about specific types of digital health interventions, caregivers’ most preferred option (66/161, 41.0% rated first choice) was receiving supportive/motivational parenting messages via SMS text message. The second highest was viewing supportive/motivational parenting posts on social media platforms (36/161, 22.4% rated first choice), followed by private groups on Facebook connecting caregivers of justice-involved youth with one another (16/161, 9.9% rated first choice). Caregivers were also willing to participate in an online support community (78/168, 46.4%), Facebook groups connecting caregivers with mental health professionals (76/168, 45.2%), and video-based sessions for individual therapy (50/168, 29.8%), family therapy (44/168, 26.2%), or support group meetings with other caregivers (40/168, 23.8%).

### Qualitative Interview

#### Sample Characteristics

The subset of 18 caregivers was 67% (12) female, on average 45 years old (SD 10), and racially/ethnically diverse (black: 10/18, 56%; white: 7/18, 39%, Latinx: 1/18, 6%; and other: 2/18, 11%). Caregivers were all familial and largely biological (16/18, 89%) parents to justice-involved youth who were on average 16 years old (SD 3). At the time of the interview, 56% (10/18) of justice-involved youth had ongoing court appointments, 50% (9/18) were on probation, 33% (6/18) had pending charges, and 6% (1/18) were detained.

#### Technology and Social Media Usage

All caregivers owned a smartphone/tablet, and most had regular computer access (15/18, 83%). Caregivers endorsed using multiple social media platforms, most commonly Facebook (18/18, 100%), Instagram (13/18, 72%), and Twitter (11/18, 61%). Snapchat and WhatsApp were used by less than 35% of the sample. Most caregivers (17/18, 94%) reported using social media to connect with family and friends, with few (4/18, 22%) connecting with people they met online. Half of the caregivers endorsed online support group membership, with only one indicating this group was specifically for parents of children on probation.

#### Digital Health Intervention Vignettes

Caregivers were highly receptive to participating in the hypothetical digital health interventions. Perspectives on each proposed intervention are presented in order of preference.

### Video Family Therapy

Almost all caregivers (17/18, 94%) reported they would participate in video-based family therapy, primarily because of convenience. Some liked that sessions could work around a caregiver’s schedule balancing work and family needs (n=4), whereas others liked saving time through eliminating the need for transportation (n=9):

I like the fact that it could be in home. You know, if I’m having a really tough day, like we can do a session on Skype or, you know, I really like that it could come both ways without having to go to the office and... just fit in with the lifestyle of a busy person who has a family and a whole lot going on because 24 hours in a day might seem large but it’s so small when you have to cram an hour here, an hour there, hour here, hour there, four hours here, six hours there. It just seems like it would help a lot.

Native American female, 32 years

Some liked the idea of video-based family therapy because it would deliver professional support or treatment from which their youth could benefit (n=5). Others liked that video-based family therapy would expand the reach of mental health services to under-resourced populations (n=2):

[D]ispersing information on a wide scale, it would be great. You could affect a great community, you know, at one time. Everybody having somewhat the same concerns. You can reach a greater audience as opposed to scheduling appointments.

Black male, 56 years

Only one caregiver stated they would not participate in video-based family therapy, specifically because he was not computer savvy (black male, age 55 years). However, most participants (n=10) noted aspects of this modality they disliked. In all, 5 of 6 males expressed concerns, compared with 5 of 12 females. Males expressed a range of dislikes, yet females primarily expressed concerns about the therapist’s credibility and their youth’s willingness to participate. Women of color expressed concerns about being able to trust the therapist leading the session is reliable or credentialed (n=3):

I guess like knowing whether or not the person is legit or not. I don’t know, like when you go to a doctor’s office they have all their credentials on the wall, you know... what if it was just some person you didn’t know but was pretending to be?

Multiracial female, 36 years

Other concerns included how privacy would be maintained and whether sessions would be confidential (n=3), feeling like they would be stigmatized if they included extended family members in sessions (n=1) and a loss of intimacy between the therapist and the client because of the use of a video platform (n=1):

...to me, video is fine for informal conversation, casual conversation. Some people use it for business communication, but again, when you’re dealing with somebody’s health or somebody’s life, I think that there’s a lot that could be lost, because in dealing with lives you want the best opportunity.

Black male, 56 years

Caregivers mentioned several barriers to participating in video therapy sessions. Technological barriers included the potential of losing access to the internet connection (n=2) and concerns about ease of use (n=1). Caregivers expressed concerns about scheduling, related to their own availability (n=3), provider availability (n=1), and after-hours support (n=1). Other barriers included financial costs (n=1) and distractions at home (n=1). Some caregivers mentioned their youth’s willingness to participate would be the biggest barrier (n=4):

I think, honestly, it would work for me as the parent of the juvenile, my son, but I feel like my son would not do it. I feel like video chat and him sitting down? He won’t do it... I know my son won’t do it and even if I did get my son to sit down and try and do it, he wouldn’t talk.

Black female, 38 years

### Private Facebook Group

Most (16/18, 89%) caregivers said they would participate in a private Facebook group to connect with other caregivers of justice-involved youth and a mental health professional. Almost all (n=15) caregivers agreed providing a space for social support made the group highly appealing:

Maybe, you know, there’s helpful ideas or even just like I said, having somebody to talk to, knowing somebody is going through—going through, or have gone through what you’ve gone through before. It’s good. I mean, I had positive experience with it—with the miscarriage group because I didn’t know anybody that had had one. And I didn’t—I didn’t know how to deal with it—what to do, you know? And when you connect with people that are going through the same thing or have gone through it, like sometimes you just need to hear that, you know, to feel better. Like okay, I’m not the only one.

White female, 35 years

Many (n=12) caregivers shared they would like to participate in the group because it offers the opportunity to speak to a mental health professional—someone who can serve as a credible resource for knowledge acquisition about mental health care. Half (n=9) of the caregivers expressed a preference for communicating with the mental health professional through video rather than phone or chat/Facebook messenger:

You can learn a lot from a person just by looking at them and seeing them, you know, see if you believe in them. Again, it’s all through the eyes. But, yeah, in the beginning, absolutely and on video. Once you get to know them and you’re pretty comfortable with them and their words resonate with you, then sure, then you can scale it back a little bit and do, you know, instant messaging, you know, things like that next, what have you, emails.

Latinx male, 50 years

More than half (n=10) of the caregivers liked that their friends and family would never know they are a part of the group because of Facebook privacy settings. A primary concern, however, was whether they could trust the other caregivers to maintain privacy (n=8) as well as personally needing time to build trust (n=4):

Because I don’t know who any of these people are. So it’s like if I post something that I wanted to be within that group of members, I don’t want to see something like screenshotted or any of my information exposed to everybody on the universe of Facebook or any type of social media group.

White female, 37 years

Several caregivers expressed concerns about other caregivers being unsupportive of the group as a unit (n=1), dominating conversations (n=1), and judging or attacking other group members (n=4). Caregivers (n=4) suggested a moderator would be useful to manage the group and remedy some of these concerns. Caregivers noted other potential barriers, including availability in their own schedules (n=5); issues with technology, specifically fearing Facebook hacks (n=1); losing internet connection (*n*=1); feeling as though online groups lack intimacy (n=1); and preferring in-the-moment responses (n=1).

### Text Messaging Intervention

Most interview participants (14/18, 78%) stated they would enroll in a text messaging intervention where they receive appointment reminders and supportive/motivational parenting messages. Caregivers identified the primary benefit as the convenience of receiving reminders about appointments (n=14):

I think they should have had this all along. I think that would—this would—would help thousands of people in the system going through different things. I think it’s great. I think, uh, that would eliminate a lot of missed - missed show ups at court. A lot of times people, you know, have issues or forget, oh, I thought it was this date, that date. I think it’s great. I think it would manage a lot of people’s families and help folks a lot better.

Black female, 47 years

Caregivers liked that the system was technology based, rather than on paper (n=3), and several caregivers shared they receive similar reminders through a current health care provider and have found these beneficial (n=3):

Oh, the reminders have been lifesaving. I mean, it has totally saved me in getting to an appointment. It has totally reminded me of what the specific—specificity of the appointment was for. And so it’s allowed me to make that appointment and get there on time and be prepared. So I think it’s a great benefit.

Black male, 56 years

Several caregivers, predominantly female, also expressed concerns. Some participants felt the reminders were unnecessary (n=3), instead preferring just the supportive/motivational messages (n=1). Some also had concerns about receiving too many text messages (n=3) or their own availability to see and respond to the messages (n=3).

Caregivers shared concerns about whether the system would function properly, including whether (1) information (eg, appointment times) would be accurate (n=1), (2) the system would update for rescheduled appointments (n=1), (3) the alert would go through on their phone (n=1), and (4) they would be properly removed after service use ended (n=1). Additional privacy concerns included who would be managing the system and the type of information they would have access to (n=1), having identifying information in text messages (n=1), or having messages pop-up that others could see (n=1):

I would wonder who’s managing that type of stuff and what other things can access through that information...Like are they just going to know that there is an appointment that day, or are they going to know what it’s about, or have any details of the case? Just making sure that that type of information is secure.

Multiracial female, 36 years

Several caregivers noted technology could be a barrier, including their phone not working consistently (n=2), having limitations to one’s data/texting plan (n=1), and disliking the use of text messaging (n=1).

## Discussion

### Main Findings

This mixed methods study examined the feasibility of recruiting caregivers of justice-involved youth into research through social media and their perceptions regarding the acceptability of digital health interventions. The results suggest recruitment of this population through Facebook is highly feasible, and caregivers of justice-involved youth are receptive to a wide range of digital health interventions.

Recruitment was highly successful, yielding a diverse sample. Within 3 weeks, more than 3000 individuals clicked the advertisement link; cost per click was US $0.53, conversion rate was 11.5%, eligibility was 43%, and cost per participant was US $10.73. Our success was comparable with other studies, where the median cost per click for advertisements was US $0.51, conversion rate was 4% (range 0.06-29.50), eligibility was 61% (range 17-100), and cost per participant was US $14.41 [39]. This is promising, given justice involvement continues to be stigmatized, making in-person recruitment challenging. Furthermore, the cost per participant is much lower than that of in-person recruitment, which requires significant staff time; this lends promise to collecting data from diverse samples even when under budgetary constraints, as with this study.

Caregivers who participated in the survey and interview were open to participating in digital health interventions and expressed a wide range of preferences. More than half of survey respondents reported they would definitely or probably participate in a digital health intervention for caregivers of justice-involved youth. The most preferred option was a text messaging intervention that provides regular supportive/motivational parenting messages directly to their mobile phone. Caregivers’ least preferred interventions were video-based individual or family therapy. In contrast, qualitative interview participants preferred video-based family therapy over the text messaging intervention. It is possible the additional details provided during the qualitative vignettes about each proposed intervention resulted in more openness to participating in the video-based therapy. Alternatively, those who elected to participate in the telephone interview might be more open to interventions involving reciprocal communication with a professional.

Qualitative interview participants endorsed many positive attitudes toward possible digital health interventions. Caregivers predominantly liked the hypothetical interventions because they could benefit from social support from peers with similar experiences, professional support from a licensed clinician, or both. They also liked that the digital delivery of interventions could resolve barriers to accessing care, primarily related to transportation and difficulty scheduling. Despite these noteworthy benefits, caregivers also shared concerns. Primary concerns surrounded privacy and information sharing, especially regarding their youth, and willingness of their youth to participate in the interventions. Concerns were also raised about technology’s functionality and reliability, the credibility of the involved mental health professional, and behavior of other participants in group-based interventions.

### Strengths and Limitations

This study has several notable strengths and limitations that can guide future research. Strengths include the mixed methods approach, nationwide sampling, and diversity in the caregivers recruited in terms of age, gender, and race/ethnicity. Obtaining perspectives from diverse caregivers through multiple methods allowed for a more comprehensive (ie, nationwide survey) and nuanced (ie, in-depth interviews) investigation of the use of mHealth technology to recruit and intervene with caregivers of justice-involved youth. The study provides support for using social media to engage caregivers of justice-involved youth in both research and treatment, as well as several possible acceptable avenues for intervention. The use of these strategies and proposed interventions has the potential to expand these families’ access to treatment and promote health equity.

As with most internet-based data collection, a key study limitation involves verifying respondent identity. We relied on self-report of status as a caregiver of a justice-involved youth, so it is possible some respondents mischaracterized themselves to gain study entry. Given the incentive was minimal and was only provided for those who completed the full survey, it seems unlikely this would have motivated individuals to falsely identify themselves as caregivers of justice-involved youth. We were also not able to track internet protocol (IP) addresses, so it is possible some respondents attempted the survey more than once; we combatted this by removing cases where the email was identical for multiple surveys (very small percentage of cases). Future research should consider requesting the verification of identity and youth’s justice involvement through official records, although this is highly sensitive information and given mistrust of researchers is common, this could limit the willingness of caregivers to participate. When possible, tracking IP addresses in future studies could provide an additional method of preventing multiple entries from the same individual.

Our study also relied on recruitment from a single social media platform, so we did not reach caregivers who use social media platforms at the exclusion of Facebook. We used Facebook because in the United States, 75% of parents use Facebook [40], and approximately one-third of Facebook users are between the ages of 35 and 54 years, the age of most caregivers of justice-involved youth [41]. Furthermore, sociodemographic characteristics of participants recruited through Facebook tend to mirror those recruited through more traditional methods or national statistics [39], and with the rise of *smart* mobile devices, racial/ethnic and economic disparities in social media use have decreased substantially [42]. Facebook also offers an easy to use advertisement platform through which to conduct research. Future studies should consider expanding recruitment to other social media sites (eg, Twitter) to reach a wider range of caregivers.

### Future Directions

The results of this study suggest recruiting and intervening with caregivers of justice-involved youth through social media is a feasible and acceptable approach. Caregivers expressed willingness to participate in a wide range of digital health interventions. Their preferences and concerns varied, however, suggesting the need for a range of interventions to increase access to care for this population. Work is underway to develop and evaluate a text messaging system that provides appointment reminders and motivational messages [29] as well as to adapt an existing in-person family-based intervention [13] to be delivered via telehealth. In future development of social media–based interventions, researchers and practitioners should consider ways to address privacy concerns (eg, security and type of platform), use of a moderator to manage group-based intervention discussions, and ways to demonstrate practitioner credibility (eg, proof of licensure). Interventions involving a clinician or moderator may also require nontraditional work hours, as many caregivers communicated the benefit of scheduling flexibility as they juggle competing demands.

## Appendix

Multimedia Appendix 1 Advertisement testing.

## Abbreviations

IP: internet protocol
mHealth: mobile health
