# Supplementary material for: Feasibility of Social Media–Based Recruitment and Perceived Acceptability of Digital Health Interventions for Caregivers of Justice-Involved Youth: Mixed Methods Study
Source: J Med Internet Res. 2020 Apr 30;22(4):e16370. doi: 10.2196/16370 (PMC7226029; doi:10.2196/16370)
Supplement: Multimedia Appendix 1 [file jmir_v22i4e16370_app1.docx]

# Multimedia Appendix

#### Advertisement Testing

Advertisement design was determined using Facebook A/B split-testing comparing four pre-selected images (stock photos) paired with the text (Figure 1), “Is your child (ages 10-17) involved in the justice system? Receive a $15 gift card for completing a brief, confidential survey for the University of California, San Francisco.” Advertisements were presented to adults: 1) ages 28 years and over; 2) using Facebook in English; and 3) residing in 10 U.S. metropolitan areas (New York/Newark/Jersey City; Los Angeles/Long Beach/Anaheim; Chicago/Naperville/Elgin; Dallas/Ft. Worth/Arlington; Washington/Arlington/Alexandria; Houston/The Woodlands/Sugar Land; San Francisco/Oakland/Hayward; Philadelphia/Camden/Wilmington; Boston/Cambridge/Newton; Atlanta/Sandy Springs/Roswell). Split test advertisements ran for three days with a maximum daily budget of $80. The advertisement with the lowest cost per link click at the end of the split test was used for the primary campaign.

*
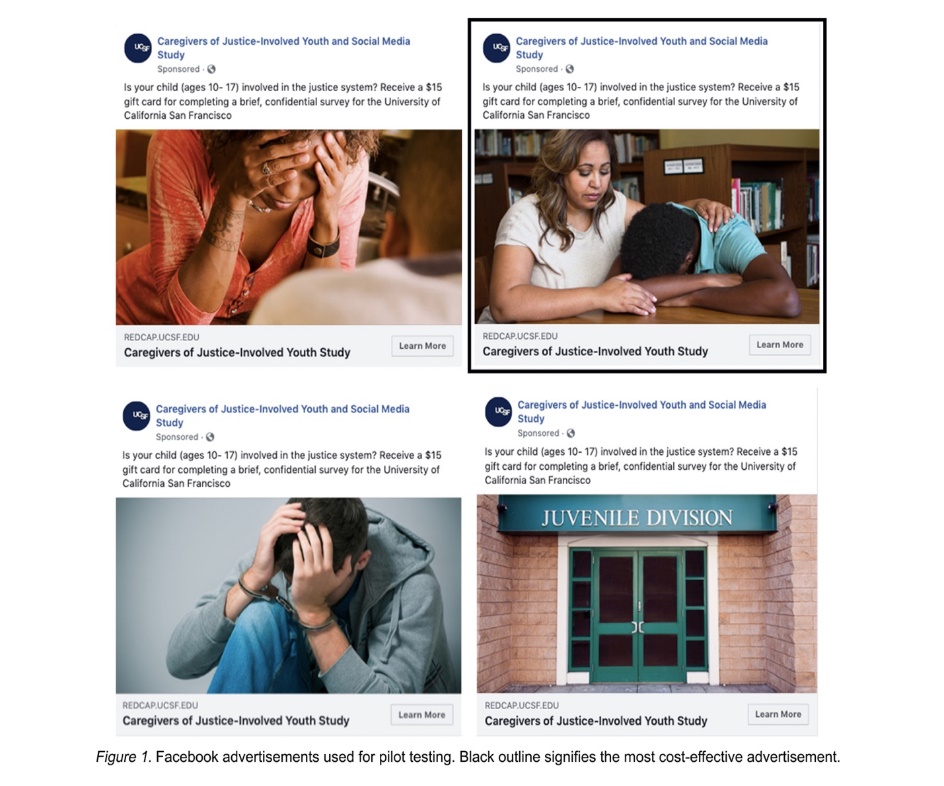
*

*Figure 1.* Facebook advertisements used for pilot testing. Black outline signifies the most cost-effective advertisement.
